# Correlation between the RNA Expression and the DNA Methylation of Estrogen Receptor Genes in Normal and Malignant Human Tissues

**DOI:** 10.3390/cimb46040226

**Published:** 2024-04-19

**Authors:** Ju Rong, Xiaojun Xie, Yongdong Niu, Zhongjing Su

**Affiliations:** 1 The First Clinical Institute, Shantou University Medical College, Shantou 515041, China; 2 Department of Histology and Embryology, Shantou University Medical College, Shantou 515041, China; 3 Department of Pharmacology, Shantou University Medical College, Shantou 515041, China

**Keywords:** estrogen receptor, expression profile, DNA methylation, carcinoma

## Abstract

Estrogen plays a multifaceted function in humans via interacting with the estrogen receptors ERα, ERβ, and G protein-coupled estrogen receptor 1 (GPER1). Previous research has predominantly concentrated on elucidating the signaling route of estrogen. However, the comprehensive understanding of the expression profile and control of these estrogen receptors in various human tissues is not well known. In the present study, the RNA levels of estrogen receptors in various normal and malignant human tissues were retrieved from the human protein atlas, the cancer genome atlas (TCGA), and the genotype-tissue expression (GTEx) databases for analyzing the expression profile of estrogen receptors through gene expression profiling interactive analysis (GEPIA). The status of DNA methylation of estrogen receptor genes from TCGA were analyzed through the software Wanderer and cBioPortal. The MethSurv tool was utilized to estimate the relevance between specific cytosine–guanine (CG) methylation and tumor survival. The expression profile analysis revealed that ERα, ERβ, and GPER1 have unique expression patterns in diverse tissues and malignancies. The interesting results were the higher expression of ERβ RNA in the male testis than in females and the positive association between the RNA level of ERα and the androgen receptor in different human normal tissues. Especially, the significant changes in GPER1 expression in multiple malignancies showed a consistent decrease with no exception, which indicates the role of GPER1 in common tumor inhibition. The finding on the expression profile provides clues for exploring novel potential physiological and pathophysiological functions of estrogen. The DNA methylation analysis manifested that the expression of GPER1 and ERα showed a substantial correlation with the methylation of specific CG sites in the cis-regulating region of the gene. However, no such association was observed for ERβ. When comparing tumor tissues to normal tissues, the DNA methylation of certain CG sites of estrogen receptors showed a correlation with tumor survival but did not always correlate with the expression of that gene or with the expression of DNA methyltransferases. We proposed that the variation in DNA methylation at different CG sites in estrogen receptor genes had other functions beyond its regulatory role in its gene expression, and this might be associated with the progression and therapy efficiency of the tumor based on the modulation of the chromatin configuration.

## 1. Introduction 

Estrogen is the primary sex hormone in females and is present at relatively low levels in males. The hormone has several functions in reproduction, tumor formation, immunological activity modulation, and cardiovascular disease development [1,2,3]. Traditionally, estrogen signals are transmitted to the nucleus by attaching to nuclear receptors ERα and ERβ, which are coded by the estrogen receptor 1 (*ESR1*) and estrogen receptor 2 (*ESR2*) genes, respectively. Upon binding to estrogen-related ligands, ERα and ERβ form homodimers or heterodimers that interact with specific DNA sequences to control the transcription of genes that are targeted by estrogen. In addition to interacting with classic nuclear receptors, estrogen can also bind to membranous G protein-coupled estrogen receptor 1 (GPER1) located on the cell membrane [4,5]. GPER1, commonly referred to as GPR30 (G protein-coupled receptor 30), is predominantly situated on the membrane of the endoplasmic reticulum and mitochondria, where it functions in fast nongenomic signaling [6,7]. The presence of the membranous estrogen receptor contributes tremendously to the functional versatility of estrogen.

Various functions of estrogen are mediated by different types of estrogen receptors, which bind to a range of exogenous and endogenous forms of estrogen. Estradiol (E2) is a highly potent female sex hormone that has a strong ability to bind to estrogen receptors ERα, ERβ, and GPER1. It plays a crucial role in the development of female secondary sexual characteristics. Additionally, it explains why autoimmune diseases are more prevalent in females compared to males [8,9]. Unlike E2, estriol (E3) exhibits a higher affinity for ERβ compared to ERα. It has been utilized in the treatment of the autoimmune disease multiple sclerosis due to its ability to regulate the balance between Treg and Th17 cells and provide neuroprotection [10,11,12]. The example illustrating the contrasting signaling roles of E2 and E3 highlights the significance of comprehending the expression profile and regulation of estrogen receptors in various tissues for a comprehensive grasp of the intricate pathophysiological function of estrogen. Despite several studies elucidating crucial pathways in certain cells or tissues, there are still unresolved problems about the transcriptional regulation of estrogen receptors.

The present investigation gathered transcriptome and epigenome data on the estrogen receptor genes *ESR1*, *ESR2*, and *GPER1* from various normal and malignant human tissues, which were obtained from online biological databases. By evaluating these data, we examined the expression characteristics of estrogen receptors in different human organs and investigated potential factors that can influence the expression of these receptors.

## 2. Methods

### 2.1. Data Retrieval and Analysis of RNA Expression of Estrogen Receptors in Normal Tissues

The RNA levels of the primary sex hormone receptors, including estrogen receptors, progesterone receptor (PGR), and androgen receptors (ARs), in normal human tissues were obtained from the human protein atlas (HPA) project’s dataset on normal tissue RNA-Seq (Accession: PRJEB4337), which contains tissue samples from 95 human individuals, representing 27 distinct tissues [13]. The Pearson index was used to analyze the correlations between the RNA levels of ESR1, ESR2, GPER1, PGR, and AR.

### 2.2. Comparing the Expression of Estrogen Receptors in Tumor and Normal Tissues

The RNA levels of estrogen receptors in tumor tissues and patient clinical information were analyzed through the large-scale gene expression profiling interactive analysis [14], based on 9736 malignancy and 8587 normal samples from the cancer genome atlas (TCGA) and the genotype-tissue expression (GTEx) databases, and the R (version 3.3.2) and Perl (version 5.22.1) programs were used to develop the plotting features. When analyzing the expression of estrogen receptors in tumor and normal tissues, the log2 (transcripts per million + 1) was used to scale the expression values, and the comparation was based on setting Log2FC = 1.

### 2.3. Analysis of DNA Methylation of Estrogen Receptors and Its Correlation with RNA Levels

The software Wanderer (http://maplab.imppc.org/wanderer, accessed on 13 April 2024) was used to visualize the methylation of cytosine–guanine (CG) sites in the estrogen receptor. Wanderer, an interactive web viewer that enables the extraction and interpretation of DNA methylation and RNA expression data from various human malignancies [15], was used to visualize the methylation of CG sites in the estrogen receptor. The DNA methylation meta-value from TCGA database was based on the Illumina HumanMethylation450 (HM450) BeadChip (data from Illumina HiSeq RNAseq not included). The methylation probe that exhibited the highest level of negative correlation with RNA levels was examined using cBioPortal (https://www.cbioportal.org, accessed on 13 April 2024) [16] for genes that have multiple methylation probes. The Pearson index was used to analyze the correlations.

### 2.4. Estimating the Relevance between CG Methylation and Tumor Survival

The MethSurv tool (https://biit.cs.ut.ee/methsurv, accessed on 13 April 2024) [17] was used to determine the link between CG methylation and tumor survival. MethSurv utilizes the Cox proportional hazard model to perform survival analysis based on TCGA cancer datasets (March 2017). The clinical and clinicopathological characteristics of patients were appropriately matched in this research. The tumor samples were split by the mean, and the Pearson index was used to analyze the correlations.

### 2.5. Statistical Analysis

The data were analyzed using GraphPad Prism 6.02 or software mentioned above. The differences were considered statistically significant when *p* < 0.05. An absolute value of the Pearson correlation coefficient (R) greater than 0.5 was defined as strong correlation, 0.3 to 0.5 as moderate correlation, and less than 0.3 as weak correlation.

## 3. Results

### 3.1. Expression Profiles of Estrogen Receptors in Normal Tissues

**Figure 1:**
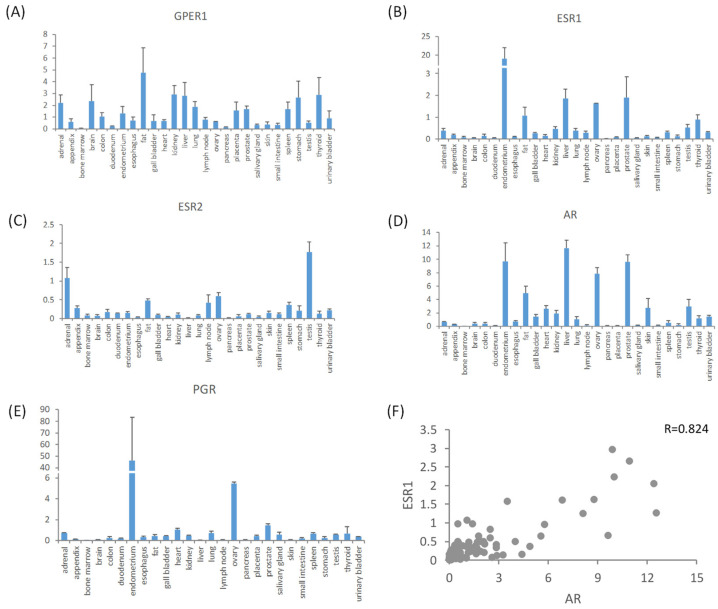
The RNA levels of the main sex hormone receptors in normal human tissues. The panel indicates the RNA levels of estrogen membrane receptor GPER1 (**A**), estrogen nuclear receptor ESR1 (**B**), estrogen nuclear receptor ESR2 (**C**), androgen receptor AR (**D**), and progesterone receptor PGR (**E**), and the correlation between the RNA level of AR and ESR1 (**F**). RPKM: reads per kilobase of exon model per million mapped reads.

The RNA levels of ESR1, ESR2, and GPER1 were obtained from the HPA in order to investigate the expression pattern of the estrogen receptors. According to the investigation, the RNA of the estrogen membrane receptor GPER1 was present in the majority of normal human tissues (Figure 1A). Classic estrogen nuclear receptor ESR1 RNA was expressed at a prominent high level in the uterine endometrium, which was approximately 10-fold more than the levels in other highly expressed tissues, including prostate, liver, ovary, and fat (Figure 1B). ESR2 RNA was expressed at the highest levels in the testes followed by the adrenal gland > ovary > fat > lymph node > other tissues. (Figure 1C). The expression level of ESR1 RNA in different human normal tissues showed a positive association with the expression level of AR RNA (Pearson correlation = 0.824, after excluding the extraordinarily high values observed in the uterine endometrium). However, there was no significant link observed among other sex hormone receptors (Figure 1D–F).

### 3.2. Expression Features of Estrogen Receptors in Malignant Tissues

**Figure 2:**
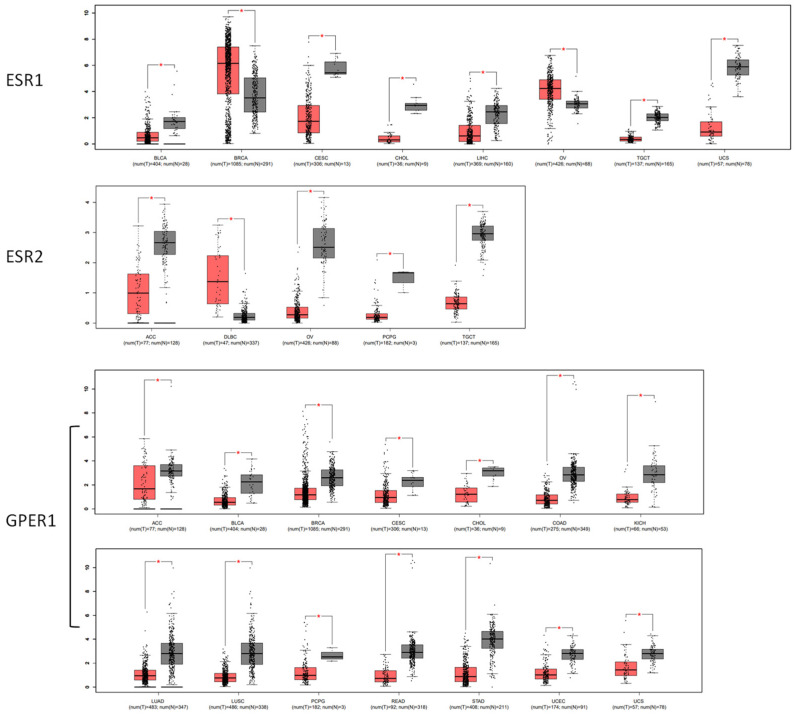
The significantly varied RNA levels of estrogen receptors ESR1, ESR2, and GPER1 in malignant tissues (indicated in red) compared to normal tissues (indicated in gray) in TCGA and GTEx. * *p* < 0.05.

Based on the data of 38 human tumor types from TCGA and the GTEx, the tumors with significantly altered expression of estrogen receptor are presented in Figure 2. The analysis revealed that, compared to normal tissues, the levels of ESR1 RNA were significantly decreased in bladder urothelial carcinoma (BLCA), cervical squamous cell carcinoma and endocervical adenocarcinoma (CESC), cholangiocarcinoma (CHOL), liver hepatocellular carcinoma (LIHC), testicular germ cell tumors (TGCTs), and uterine carcinosarcoma (UCS). On the other hand, breast invasive carcinoma (BRCA) and ovarian serous cystadenocarcinoma (OV) showed increased expression of ESR1 RNA.

The levels of ESR2 RNA were reduced in adrenocortical carcinoma (ACC), ovarian cancer (OV), pheochromocytoma and paraganglioma (PCPG), and testicular germ cell tumors (TGCTs). The expression of ESR2 RNA was elevated in diffuse large B-cell lymphoma (DLBC).

The expression of GPER1 RNA was found to be reduced in several types of cancer, including adrenocortical carcinoma (ACC), bladder urothelial carcinoma (BLCA), breast invasive carcinoma (BRCA), cervical squamous cell carcinoma and endocervical adenocarcinoma (CESC), cholangiocarcinoma (CHOL), colon adenocarcinoma (COAD), kidney chromophobe (KICH), lung adenocarcinoma (LUAD), lung squamous cell carcinoma (LUSC), pheochromocytoma and paraganglioma (PCPG), rectum adenocarcinoma (READ), stomach adenocarcinoma (STAD), uterine corpus endometrial carcinoma (UCEC), and uterine carcinosarcoma (UCS). No notable elevation of GPER1 RNA was observed in any of the tumors in comparison to normal tissue.

### 3.3. The DNA Methylation Profile of Estrogen Receptor Genes

**Figure 3:**
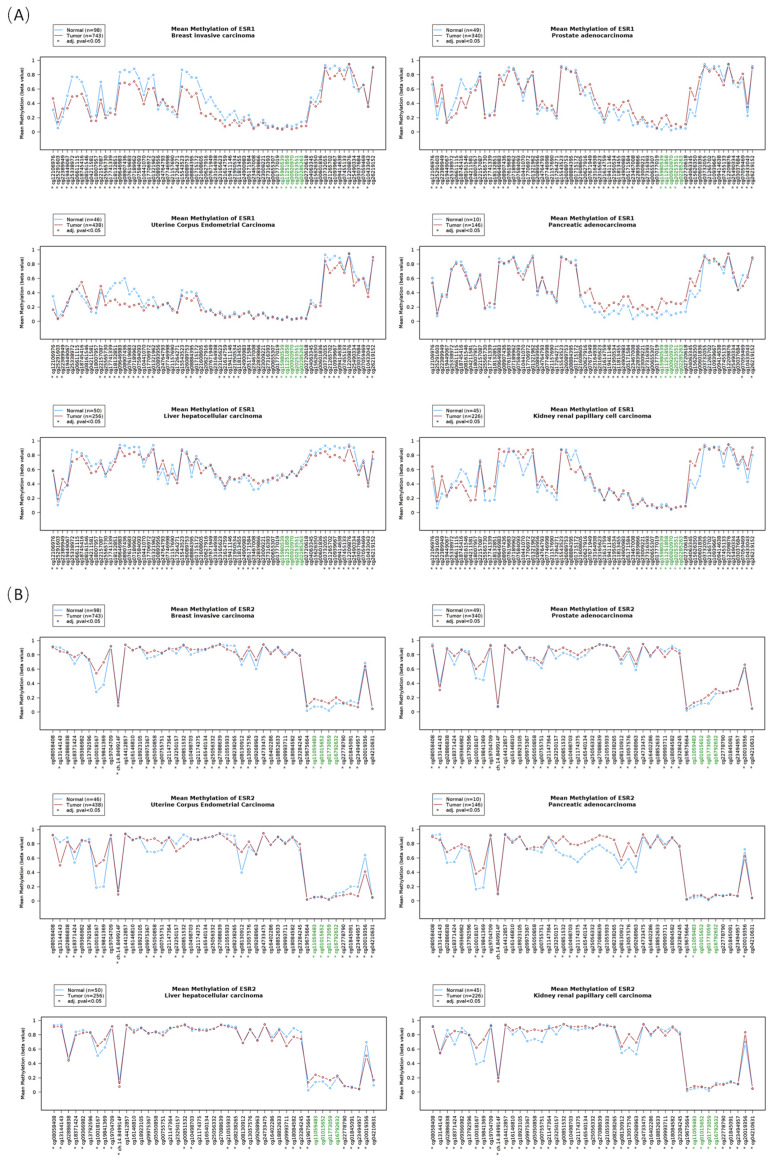

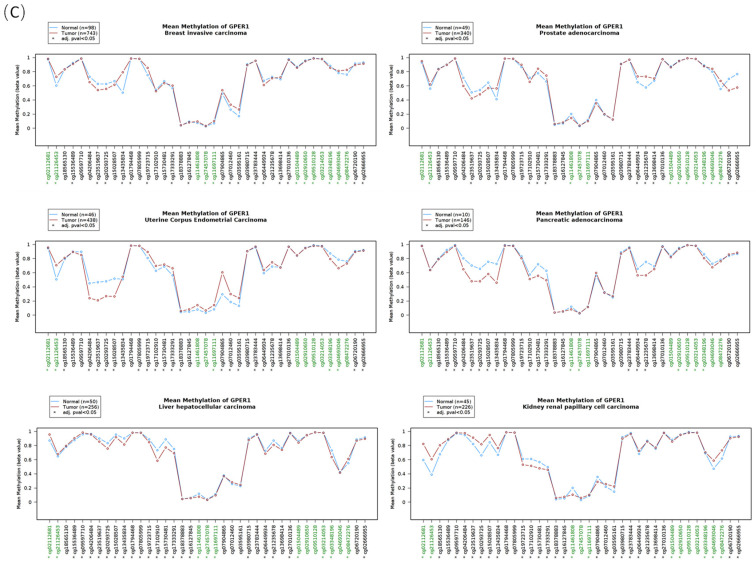
The DNA methylation profile of the estrogen receptor genes *ESR1* (**A**), *ESR2* (**B**), and *GPER1* (**C**) in malignant tissues compared to normal tissues (* *p* < 0.05). The CG probes of DNA methylation were presented uniformly to clearly show the CG sites. Green indicates the CG islands.

Using the Wanderer software, the DNA methylation of estrogen receptor genes in tumor and normal tissues was analyzed. The representative DNA methylation profiles of these three receptor genes *ESR1*, *ESR2*, and *GPER1* in male prostate cancer, female breast invasive carcinoma, uterine corpus endometrial carcinoma, pancreatic adenocarcinoma, hepatocellular carcinoma, and renal papillary cell carcinoma are listed in Figure 3. The findings show that each receptor gene’s DNA methylation patterns were consistent across various samples. In addition, upon comparing the tumor tissues with normal tissues, notable alterations, including both increased and decreased methylation, were observed at different CG sites (*p* < 0.05).

The DNA methylation profile also provides information about the methylation status of CG islands in various regions of the gene. Based on blasting the DNA sequence, we found that the CG islands located in the promoter region of *ESR1* (the only CG island in Figure 3A) and *GPER1* (the second CG island in Figure 3C) were relatively hypomethylated overall, though there were some differences for each CG site methylation between normal and tumor tissues. The CG islands located in the intergenic or intragenic region of *GPER1* (the first and third CG island in Figure 3C) exhibited a relatively high level of methylation, while the CG island in the intragenic region of *ESR2* showed a lower level of methylation (the only CG island in Figure 3B), and there was no CG island in the promoter region of the *ESR2* gene.

**Figure 4:**
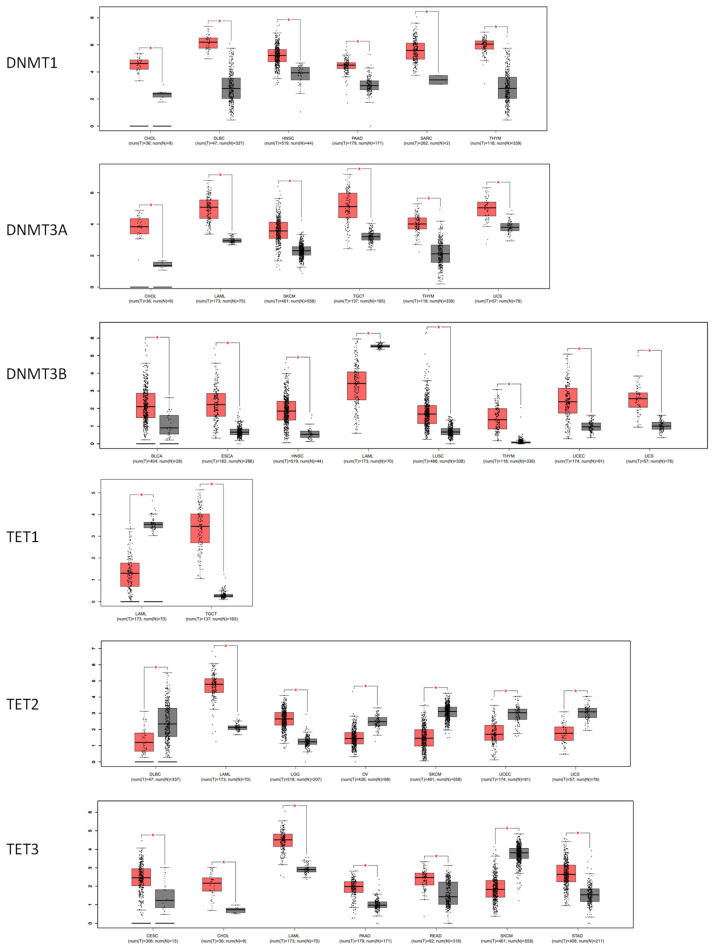
The significantly varied RNA levels of DNA methyl transferases DNMTs and TETs in malignant tissues (indicated in red) compared to normal tissues (indicated in gray) in TCGA and GTEx. * *p* < 0.05.

The DNA methyltransferases (DNMT1, DNMT3A, and DNMT3B) and dioxygenase (TET1, TET2, and TET3) play crucial roles in the process of DNA methylation. The GEPIA study reveals that, when compared with normal tissues, the expression of DNMT1 is elevated in CHOL, DLBC, head and neck squamous cell carcinoma (HNSC), pancreatic adenocarcinoma (PAAD), sarcoma (SARC), and thymoma (THYM). DNMT3A had elevated levels in CHOL, acute myeloid leukemia (LAML), skin cutaneous melanoma (SKCM), TGCT, THYM, and uveal melanoma (UCS). DNMT3B expression was elevated in BLCA, ESCA, HNSC, LUSC, THYM, UCEC, and UCS. Only patients with LAML exhibited a reduction in DNMT3B RNA levels, but there was no significant drop in the expression of DNMT1 and DNMT3A in any of the tumors. For TETs’ expression, there were both increases and decreases in different tumor types (Figure 4).

### 3.4. The Correlation between the DNA Methylation and the RNA Level of Estrogen Receptors

The correlation between the DNA methylation beta-value and the RNA level of ESR1, ESR2, and GPER1 was analyzed through cBioPortal in 19 tumor types. Given that each gene is associated with multiple methylation probes, we have documented the strongest negatively linked connections in Table 1. ESR1 had an absolute R value greater than 0.5 in 5 tumor types (strong correlation), an R value ranging from 0.3 to 0.5 in 8 tumor types (moderate correlation), and an R value less than 0.3 in 6 tumor types (weak correlation). In the case of ESR2, none of the tumors exhibited an R value greater than 0.5, and the majority of tumors (16 out of 19) had an R value less than 0.3. GPER1 exhibited an R greater than 0.5 in 6 tumor types, R values ranging from 0.3 to 0.5 in 11 tumor types, and R values less than 0.3 in 2 tumor types.

### 3.5. The Correlation between DNA Methylation and Tumor Survival

**Figure 5:**
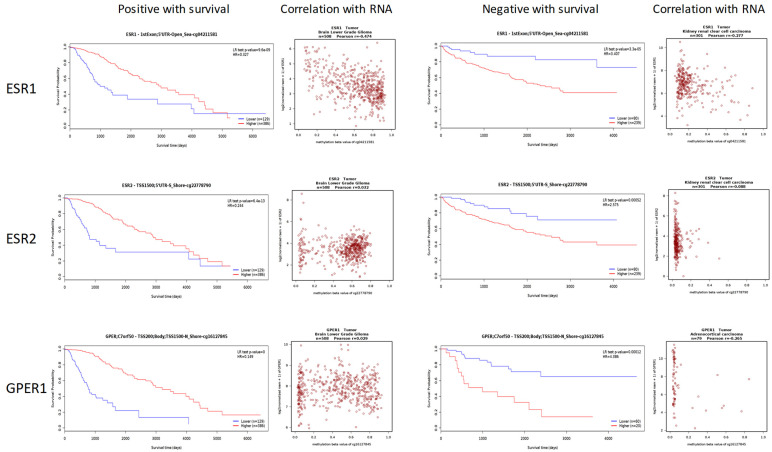
The representative CG methylation of *ESR1*, *ESR2*, and *GPER1* with contrary tumor survival and the correlation to RNA levels.

The association between DNA methylation of the estrogen receptor genes and tumor survival was examined using the MethSurv program. CG sites exhibiting a hazard ratio (HR) more than 2 and a *p*-value less than 0.05 were designated as positively correlated sites (+). Conversely, CG sites with an HR less than 0.5 and a *p*-value less than 0.05 were designated as negatively correlated sites (−). Significant associations were observed between tumor survival and ESR1 methylation (159 records, 68 negative and 91 positive), ESR2 methylation (46 records, 27 negative and 19 positive), and *GPER1* methylation (67 records, 35 negative and 32 positive). Table 2 presents the top five CG positions in estrogen receptor genes that have the highest HR values. Partial methylation of the CG sites in the genes *ESR1*, *ESR2*, and *GPER1* was found to be negatively associated with patient survival in various types of malignancies. An instance of cg17102910 hypermethylation in *GPER1* was shown to be linked to increased survival rates in lower-grade glioma (LGG) of the brain (HR = 0.197), while it was connected with decreased survival rates in ACC (HR = 13.854). Upon analyzing the connection between the methylation status of these CG sites and the RNA levels, no significant correlation (R < 0.5) was found (Figure 5).

## 4. Discussion

Estrogen hormones have intricate pathophysiological roles by attaching to the estrogen receptors ERα, ERβ, and GPER1. A previous study found that the protein expression of the estrogen receptor was consistent with the RNA-seq results in the HPA [18]. In the present study, by examining the transcriptional data obtained from 27 different types of tissues collected from 95 individuals, we discovered that ESR1 had significant expression levels in the uterine endometrium. Interestingly, this tissue also displayed the highest expression levels of the progesterone receptor. These findings indicate that the expression of estrogen receptors aligns with the primary physiological role of estrogens in controlling the growth and shedding of the uterine lining during the menstrual cycle and pregnancy [19]. In addition to the endometrium, ESR1 RNA had significant expression levels in the prostate, liver, ovary, and adipose tissues. This expression was strongly and positively linked with the levels of AR RNA (R = 0.85). Another notable characteristic is the higher expression of the estrogen receptor ESR2 in the male testis compared to the female ovary and endometrium, whereas the androgen receptor expression in the testis is lower than in the liver, ovary, and endometrium. While this is only an initial discovery, the results have led us to contemplate the potential interaction effects of estrogen and androgen in controlling the activity of these cells and organs. GPER1, the estrogen membrane receptor, is present in the majority of normal human tissues, alongside ESR1 and ESR2. We hypothesized that the widespread distribution of GPER1 in various tissues may be associated with its crucial function in regulating cell proliferation, since GPER1 has the ability to influence the signaling pathway responsible for intracellular calcium mobilization, phosphatidylinositol synthesis, and the activation of ERK1/2 and PI3K/AKT [20,21,22]. The findings regarding the expression profile of three types of estrogen receptors provide evidence that estrogen hormones, in addition to their role in the development and activity of the female reproductive system, may also interact with other hormonal signals and contribute to various physiological functions.

Previous studies have established a correlation between the presence of estrogen receptors and estrogen signaling and the development of cancers, particularly breast carcinoma [23,24,25]. We conducted a more in-depth analysis of the expression pattern of estrogen receptors in various cancerous tissues. Among the 38 tumor types in the database, 14 tumor types consistently exhibited a reduction in GPER1 RNA expression, and there was no one tumor type with significant increase in the case of GPER1. Prior research has also established the role of GPER1 in estrogen-related conditions, including female breast and ovarian malignancies [26,27], cervical and endometrial cancers [28,29], male prostate cancer [30,31], gastrointestinal tract cancers [32,33], and melanoma [34]. The decreased expression of GPER1 may be a shared mechanism associated with the development of different types of tumors, based on the signaling pathway by which it controls cell growth. The analysis of the expression pattern of estrogen receptors also offers insight into developing more precise and powerful ligands for manipulating estrogen signaling in possible therapeutic contexts.

DNA methylation is widely recognized as a crucial epigenetic factor in the control of gene expression. Researchers are highly interested in CG islands located in promoter regions because of their role in transcriptional regulation [35,36]. *ESR1* and *GPER1* exhibited CG islands in their promoter region, which displayed persistent hypomethylation. However, the analysis of the gene sequences revealed the absence of CG islands in the promoter region of ESR2. However, there is a CG island present solely within the gene body. Through the examination of the relationship between DNA methylation and RNA expression, we discovered a clear correlation between the methylation of CG sites in the promoters of *GPER1* and *ESR1* and their respective RNA expression in around one-third of tumor types. Conversely, the methylation of CG sites in *ESR2* only had a minor impact on its expression. The findings suggest that the expression of *GPER1* and *ESR1* RNA in tumor cells is probably regulated by DNA methylation. In addition to the promoter region, the DNA methylation at CG islands inside gene bodies can also modulate the gene expression by controlling the RNA polymerase II elongation rate [37,38], and the involvement of gene body methylation in the alternative splicing of RNA has been recognized in recent years [39,40]. Tumors have numerous estrogen receptor isoforms, with certain isoforms exerting a strong inhibitory effect on other members of the estrogen receptor family [41,42,43]. Currently, it is unknown whether the expression of these isoforms is associated with DNA methylation.

Apart from CG islands, the entire mammalian genome contains a substantial quantity of dispersed CG dinucleotide. Given the discovery of altered methylation patterns in various human malignancies, we were intrigued by the potential relationship between DNA methylation and tumor survival. The correlation between methylation of certain CG sites and patient survival rates is expected. The methylation of CG sites exhibited an unexpected correlation with good survival rates in one tumor, while demonstrating a correlation with low survival rates in another tumor. Furthermore, the majority of these CG sites that were associated with tumor survival did not make a meaningful impact on the gene’s RNA level. Though DNA methylation has been found to play a role in regulating gene expression, X-chromosome inactivation, gene imprinting, and centromere stability by modulating chromatin configuration [44,45], it appears that the previous results represent only the tip of the iceberg for the functions of DNA methylation. Through cross-platform data and multidimensional validations, Zhang et al. recently verified that DNA methylation at specific CG sites of *ALDH2* and *SPATS2L* gene contributed significantly to acute myeloid leukemia patient survival and immunotherapy [46]. The role of DNA methylation in promoting tumor aggressiveness through favoring tumor immune escape was also reported in adrenocortical carcinoma cells [47]. Based on the expression and function of estrogen receptors in breast cancer, anti-estrogen receptor endocrine therapy was applied for millions of breast cancer patients in clinics, while about 33% of estrogen-receptor-positive malignancies have an intrinsic potential to develop hormone resistance [48]. Though the mechanism is complex and often remains unclear, DNA methylation, together with other epigenetic factors, such as histone post-translational modifications and microRNA, were considered to be associated with the cancer cell adaptability to endocrine therapy [49,50]. The DNA methylation profile of the tumor tissues in the present study manifested that there were both increased and decreased methylation at different CG sites in the same malignancy when compared with normal tissues. We speculate that some specific variation of these CG sites’ methylation in estrogen receptor genes might connect with the tumor progression and therapy resistance, and this provides clues for designing personalized endocrine therapy strategies based on genomic signatures.

The DNA methylation is catalyzed by DNA methyltransferase DNMTs, and the TET family catalyzes the oxidation of 5-methylcytosine to 5-hydroxymethylcytosine on DNA to eraser the DNA demethylation. By analyzing the expression patterns of these DNA methylation writers and erasers, we observed that the most notable alterations in cancers were elevated levels of DNMT1, DNMT3A, and DNMT3B, which is consistent with the previous result of global DNA hypermethylation in a broad array of cancers. On the other hand, the DNA methylation profile revealed that the CG sites exhibited varying degrees of hyper- or hypomethylation in specific regions, which suggests that the local context significantly influences regional DNA methylation. However, it is undeniable that changed expression of DNMTs and TETs and mutation resulting the gain and loss of enzyme functions contribute to these processes [51,52]. When comparing one tumor with normal tissue, it was observed that the DNA methylation of estrogen receptors exhibited both increased and decreased methylation, regardless of the expression levels of the DNA methylation- and demethylation-associated DNMTs and TETs. This finding confirms that the methylation of the CG sites is mostly influenced by the local environment rather than the levels of DNMTs. Considering the intricate surrounding of the specific DNA region, including the function of epigenetic factors and natural antisense RNAs [53,54], it is crucial to exercise caution when contemplating the use of 5-Aza-2’-deoxycytidine or other drugs that target overall DNA methylation in clinical treatment. The potential impact and underlying mechanism of such therapeutic strategies should be thoroughly evaluated, particularly in cases in which the patient exhibits modified expression of DNMTs or altered levels of global DNA methylation.

In summary, through in silico analysis, the present study found that estrogen receptors ERα, ERβ, and GPER1 were expressed at different levels in diverse tissues and that this might mediate the various estrogen signaling mechanisms in cells. The most inspiring phenomena are the higher expression of ESR2 in the male testis than in the female ovary and endometrium and the consistent reduction of GPER1 RNA in different tumor samples when compared with normal tissues. Thus, the expression profile of estrogen receptors in normal and malignant human tissues provides clues for exploring novel potential physiological and pathophysiological functions of estrogen. Moreover, DNA methylation should be involved in the transcriptional regulation of ESR1 and GPER1 more than in that of ESR2, though the modulation and function of CG site methylation patterns needs further investigation.

## Figures and Tables

**Table 1:** The correlation between the DNA methylation and the RNA levels of estrogen receptors.

Tumor (Number of Patients/Samples)	ESR1	ESR2	GPER1
Adrenocortical carcinoma (88)	Pearson: −0.47 (*p* = 2.061 × 10^−5^)	Pearson: −0.49 (*p* = 7.873 × 10^−6^)	Pearson: −0.74 (*p* = 5.08 × 10^−14^)
Bladder urothelial carcinoma (127)	Pearson: −0.14 (*p* = 0.126)	Pearson: −0.18 (*p* = 0.0436)	Pearson: −0.36 (*p* = 3.259 × 10^−5^)
Breast invasive carcinoma (963)	Pearson: −0.74 (*p* = 2.52 × 10^−116^)	Pearson: −0.01 (*p* = 0.846)	Pearson: −0.36 *p* = 8.62 × 10^−22^)
Cervical squamous cell carcinoma and endocervical adenocarcinoma (191)	Pearson: −0.54 (*p* = 5.40 × 10^−16^)	Pearson: −0.05 (*p* = 0.505)	Pearson: −0.48 (*p* = 1.56 × 10^−12^)
Cholangiocarcinoma (35)	Pearson: −0.33 (*p* = 0.0551)	Pearson: −0.28 (*p* = 0.101)	Pearson: −0.67 (*p* = 9.217 × 10^−6^)
Esophageal carcinoma (184)	Pearson: −0.45 (*p* = 1.09 × 10^−10^)	Pearson: −0.09 (*p* = 0.213)	Pearson: −0.41 (*p* = 8.76 × 10^−9^)
Head and neck squamous cell carcinoma (504)	Pearson: −0.18 (*p* = 8.349 × 10^−5^)	Pearson: −0.23 (*p* = 3.90 × 10^−7^)	Pearson: −0.49 (*p* = 8.74 × 10^−31^)
Kidney chromophobe (66)	Pearson: −0.24 (*p* = 0.0557)	Pearson: −0.03 (*p* = 0.836)	Pearson: −0.47 (*p* = 7.333 × 10^−5^)
Kidney renal clear cell carcinoma (448)	Pearson: −0.41 (*p* = 3.48 × 10^−12^)	Pearson: −0.26 (*p* =2.622 × 10^−5^)	Pearson: −0.43 (*p* = 4.82 × 10^−13^)
Kidney renal papillary cell carcinoma (280)	Pearson: −0.56 (*p* = 4.33 × 10^−23^)	Pearson: −0.18 (*p* = 3.705 × 10^−3^)	Pearson: −0.63 (*p* = 1.14 × 10^−30^)
Lower-grade glioma (283)	Pearson: −0.51 (*p* = 7.10 × 10^−20^)	Pearson: −0.04 (*p* = 0.489)	Pearson: −0.26 (*p* = 1.055 × 10^−5^)
Liver hepatocellular carcinoma (366)	Pearson: 0.04 (*p* = 0.492)	Pearson: −0.34 (*p* = 2.49 × 10^−11^)	Pearson: −0.62 (*p* = 4.99 × 10^−39^)
Lung adenocarcinoma (230)	Pearson: −0.40 (*p* = 1.33 × 10^−8^)	Pearson: −0.13 (*p* = 0.0729)	Pearson: −0.38 (*p* = 8.56 × 10^−8^)
Lung squamous cell carcinoma (178)	Pearson: −0.11 (*p* = 0.344)	Pearson: −0.25 (*p* = 0.0309)	Pearson: −0.18 (*p* = 0.128)
Pancreatic adenocarcinoma (149)	Pearson: −0.31 (*p* = 1.339 × 10^−4^)	Pearson: −0.14 (*p* = 0.0982)	Pearson: −0.47 (*p* = 1.00 × 10^−9^)
Prostate adenocarcinoma (492)	Pearson: −0.36 (*p* = 2.28 × 10^−16^)	Pearson: −0.18 (*p* = 6.165 × 10^−5^)	Pearson: −0.57 (*p* = 1.00 × 10^−43^)
Thyroid carcinoma (399)	Pearson: −0.54 (*p* = 3.82 × 10^−31^)	Pearson: −0.03 (*p* = 0.548)	Pearson: −0.32 (*p* = 1.21 × 10^−10^)
Thymoma (123)	Pearson: −0.37 (*p* = 3.940 × 10^−5^)	Pearson: −0.49 (*p* = 1.86 × 10^−8^)	Pearson: −0.31 (*p* = 7.031 × 10^−4^)
Uterine carcinosarcoma (56)	Pearson: −0.18 (*p* = 0.185)	Pearson: −0.04 (*p* = 0.792)	Pearson: −0.72 (*p* = 6.00 × 10^−10^)

**Table 2:** The CG sites with the top 5 correlations between DNA methylation and tumor survival.

Name.	Cancer *	HR	CI	*p*-Value	To Ref. Gene ^#^	Island ^#^
**ESR1**						
cg25490334	UVM	0.077	(0.01; 0.578)	0.012625	Body	Open_Sea
cg08907436	KIRC	0.159	(0.07; 0.363)	1.28 × 10^−5^	5′UTR; TSS1500	N_Shelf
cg01715172	UVM	0.186	(0.043; 0.809)	0.024897	TSS1500; 5′UTR	N_Shore
cg20893956	UVM	0.231	(0.101; 0.529)	0.000518	5′UTR; TSS200	N_Shelf
cg21265702	UVM	0.232	(0.086; 0.626)	0.003911	Body	Open_Sea
cg19411146	KICH	6.807	(1.696; 27.319)	0.006824	5′UTR; 1stExon	N_Shore
cg18745416	UVM	10.649	(2.451; 46.269)	0.001599	TSS1500	Open_Sea
cg20253551	KICH	11.207	(2.325; 54.026)	0.002603	Body; 1stExon	CG Island
cg25338972	UVM	13.029	(2.995; 56.676)	0.000621	TSS1500	Open_Sea
cg06611115	UVM	20.689	(2.734; 156.579)	0.003348	TSS1500	Open_Sea
**ESR2**						
cg22778790	UVM	0.072	(0.01; 0.541)	0.010473	TSS1500; 5′UTR	S_Shore
cg23494957	UVM	0.102	(0.014; 0.759)	0.025805	TSS1500; 5′UTR	S_Shore
cg01845091	UVM	0.209	(0.049; 0.895)	0.034904	TSS1500; 5′UTR	S_Shore
cg16792632	KICH	0.237	(0.063; 0.887)	0.032475	TSS200; 5′UTR	CG Island
cg20019356	UVM	0.24	(0.105; 0.547)	0.000683	5′UTR	S_Shelf
cg23284245	UCEC	2.782	(1.576; 4.913)	0.000419	5′UTR	N_Shelf
cg01845091	ACC	2.786	(1.301; 5.965)	0.008339	TSS1500; 5′UTR	S_Shore
cg19583967	ACC	2.863	(1.293; 6.343)	0.009529	TSS1500	S_Shore
cg11059483	KICH	3.962	(1.061; 14.802)	0.0406	1stExon; 5′UTR	Island
cg19583967	UVM	4.51	(1.055; 19.285)	0.042191	TSS1500	S_Shore
**GPER1**						
cg06449934	UVM	0.083	(0.011; 0.653)	0.018014	5′UTR; Body	N_Shore
cg16127845	LGG	0.149	(0.102; 0.216)	0	TSS200; Body	N_Shore
cg18378883	LGG	0.153	(0.105; 0.223)	0	TSS200; Body	N_Shore
cg17102910	LGG	0.197	(0.136; 0.284)	0	TSS200; Body	N_Shore
cg01504489	UVM	0.227	(0.084; 0.612)	0.003393	Body	CG Island
cg07904865	UVM	5.288	(1.232; 22.694)	0.025032	5′UTR; Body	S_Shore
cg03595161	UVM	5.362	(1.243; 23.119)	0.024311	5′UTR; Body	S_Shore
cg19723715	ACC	6.093	(1.445; 25.682)	0.01382	TSS1500; Body	N_Shore
cg15730481	UVM	10.298	(1.383; 76.672)	0.022812	TSS200; Body	N_Shore
cg17102910	ACC	13.854	(1.878; 102.186)	0.009931	TSS200; Body	N_Shore

* ACC, adrenocortical carcinoma; KICH, kidney chromophobe; KIRC, kidney renal clear cell carcinoma; LGG, brain lower-grade glioma; UCEC; uterine corpus endometrial carcinoma; UVM, uveal melanoma. ^#^ UTR, untranslated region; TSS200, 200 bp upstream from the transcription start site; TSS1500, 1500 bp upstream from the transcription start site; S_Shore, up to 2 kb upstream of CG island; N_Shore, up to 2 kb downstream of CG island, S_Shelf, 2–4 kb upstream of CG island; N_Shelf, 2–4 kb downstream of CG islands; Open_Sea, remaining regions.

## Data Availability

The datasets used in this article can be acquired through the internet. They can be downloaded from the corresponding open databases mentioned in this article.
